# Aftereffect of perceived motion trajectories

**DOI:** 10.1016/j.isci.2024.109626

**Published:** 2024-03-28

**Authors:** Ryohei Nakayama, Mai Tanaka, Yukino Kishi, Ikuya Murakami

**Affiliations:** 1 Department of Psychology, The University of Tokyo, 7-3-1 Hongo, Bunkyo-ku 113-0033, Tokyo, Japan

**Keywords:** Mechanics, Applied sciences

## Abstract

If our visual system has a distinct computational process for motion trajectories, such a process may minimize redundancy and emphasize variation in object trajectories by adapting to the current statistics. Our experiments show that after adaptation to multiple objects traveling along trajectories with a common tilt, the trajectory of an object was perceived as tilting on the repulsive side. This trajectory aftereffect occurred irrespective of whether the tilt of the adapting stimulus was physical or an illusion from motion-induced position shifts and did not differ in size across the physical and illusory conditions. Moreover, when the perceived and physical tilts competed during adaptation, the trajectory aftereffect depended on the perceived tilt. The trajectory aftereffect transferred between hemifields and was not explained by motion-insensitive orientation adaptation or attention. These findings provide evidence for a trajectory-specific adaptable process that depends on higher-order representations after the integration of position and motion signals.

## Highlights

• An adaptable computational process underlies object trajectory perception
• Not physical but perceived orientations produce the trajectory aftereffect we found
• The aftereffect shows interhemifield transfer, which suggests an integrative process
• Motion-insensitive orientation adaptation or attention cannot explain the results

## Graphical abstract

**Figure:**
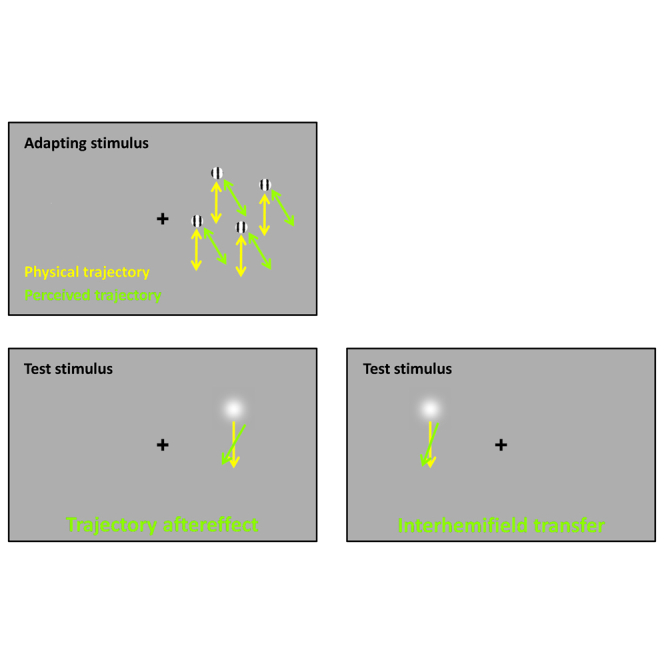

## Introduction

In natural scenes, the motion trajectories of visual objects often correlate in orientation, as seen in objects falling vertically due to gravity and moving horizontally on the ground. If our visual system is endowed with an adaptable computational process for trajectories, it may have functional significance in everyday life because it can minimize redundancy and emphasize variation in natural scene structures. Here we show psychophysical evidence for such a process.

The initial encoding of motion is known to involve dedicated architectures, such as luminance-based motion energy detectors, that act as spatiotemporal filters of luminance distributions on the retina.^1^^,^^2^ After prolonged viewing of a pattern consisting of moving dots, a static test pattern presented at the same retinal location appears to move in the opposite motion direction, consistent with the distribution-shift model of motion energy.^3^ After adaptation to a moving pattern, another pattern moving in a similar direction appears to move in a repulsive motion direction.^4^ Motion aftereffects are basically retinotopic, except for only a few cases of more complex motions than translation.^5^^,^^6^

**Figure 1:**
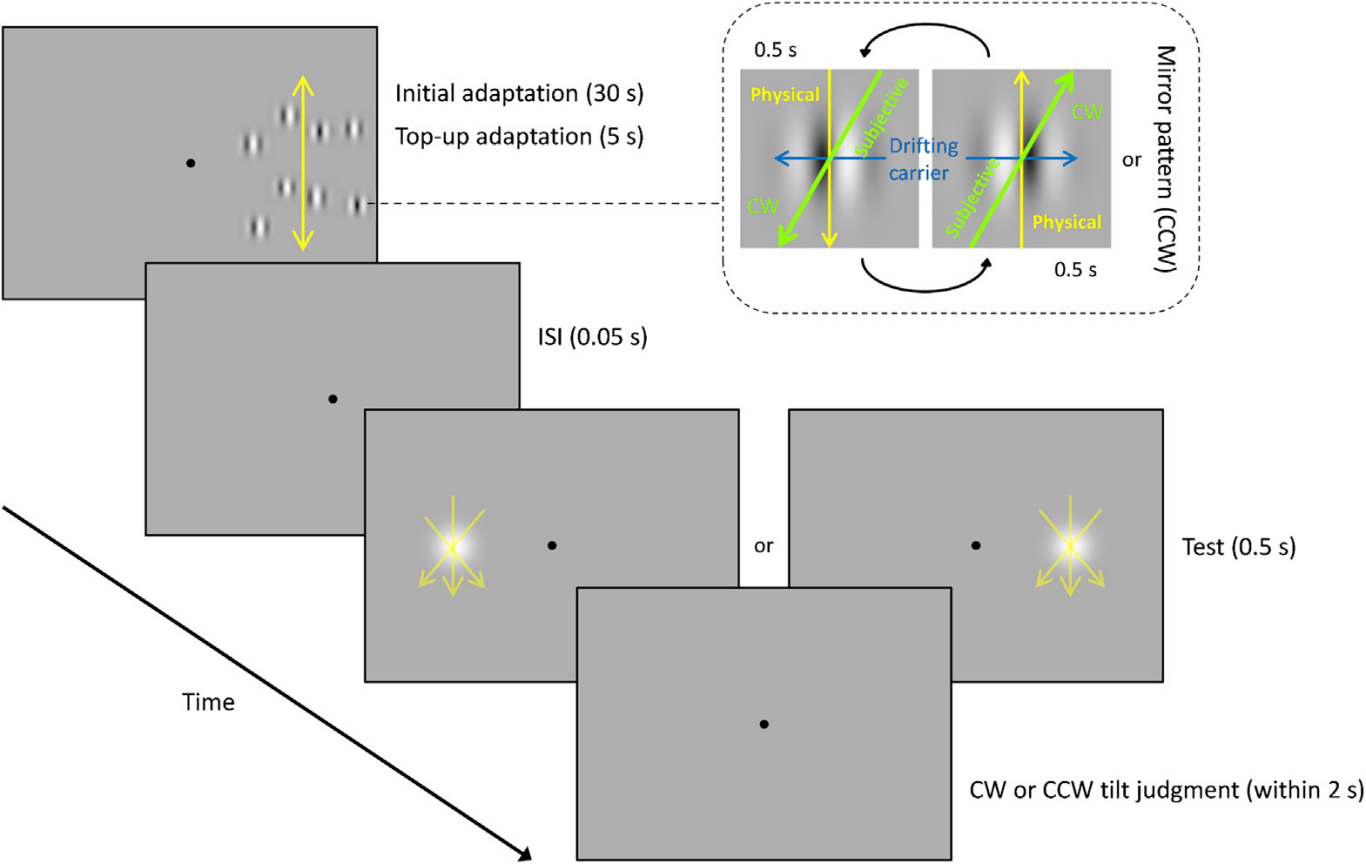
Schematic stimulus display (subjective adapt-tilt condition) In each trial, observers adapted to an array of Gabor patches, each traveling up and down and reversing the direction asynchronously with each other (depicted by the yellow arrows) but synchronously with the directional reversal of its own carrier drifting horizontally (depicted by the blue arrows). Although the patches physically traveled along the vertical axis, their motion trajectories subjectively appeared as tilting either clockwise (CW) or counterclockwise (CCW) due to the double-drift illusion (depicted by the green arrows). Adaptation lasted 35 s in the first trial of each session and 5 s in the remaining trials. To test for the trajectory aftereffect, a luminance blob was presented either within the left or right hemifield (depicted by the light-yellow arrows).

However, the perception of object trajectories depends not only on the aforementioned early motion processing but also on integrative processing stages. In the double-drift illusion, the trajectory of a traveling Gabor patch is perceived as tilting away from the veridical course, biased toward the direction of the carrier drift (Figure 1 inset).^7^^,^^8^ This bias arises as the carrier’s motion signals that normally help disambiguate the position of the Gabor patch contribute to a biased solution in the perceived position.^9^ Accordingly, the double-drift illusion is viewed as an accumulated illusion of position shifts rather than just an illusory motion^10^ and as a useful tool for delineating the relative contributions of physical and subjective trajectories to various appearances and performances.^11^

While recent evidence points to the distinct coding of trajectories from temporal integration of motion velocity,^9^ the role of visual adaptation in object trajectory perception remains unclear. Although adaptation to static orientation stimuli is known to influence the perceived orientation of a traveling object’s trajectory,^12^^,^^13^ to our knowledge, there have been no investigations on the effect of adaptation to motion trajectories, let alone subjective ones as in the double-drift illusion. In one study that examined adaptation to geometrically skewed moving images, the double-drift illusion was not always altered as predicted from an aftereffect tested with actual object motions.^14^ In the present study, we employ adaptation to the double-drift illusion, which provides an opportunity to study adaptable computational processes that may depend on physical and/or subjective trajectories.

Typically, visual aftereffects depend on adaptation to physical stimulation. While some types of motion and tilt aftereffects have been reported to occur following adaptation to illusory or implied motions^15^^,^^16^^,^^17^^,^^18^ (see studies by Fukiage and Murakami^19^^,^^20^), these aftereffects are generally much smaller than classic aftereffects after adaptation to physical features. For example, adaptation to a static upright stimulus perceived as tilting due to motion reversals induces a repulsive tilt aftereffect, but its size is approximately half that induced after adaptation to a stimulus with a physical tilt.^15^ In contrast, other visual aftereffects involving higher-order processes often exhibit weaker location specificity. For example, the face aftereffect, which emerges as an illusory repulsive distortion of facial configuration after adaptation to distorted faces, is believed to involve face-selective brain areas with little location specificity, and indeed transfers to retinally unadapted locations.^21^ Thus, examining whether and how aftereffects in question exhibit location invariance will provide constraints about possible computational stages and neural loci responsible for the aftereffects.

We found that after adaptation to multiple objects traveling along trajectories that appeared as tilting, the vertical trajectory of a single object was perceived as tilting on the repulsive side. This novel illusion (hereafter “trajectory aftereffect”) occurred to virtually the same extent, irrespective of whether the tilt of the adapting stimulus (hereafter “adapt-tilt”) was physical or only subjectively available as the double-drift illusion. Moreover, the trajectory aftereffect was induced by a subjective tilt, even in the presence of a competing physical tilt. Strikingly, the trajectory aftereffect partially transferred between the left and right hemifields. Additional experiments confirmed that the trajectory aftereffect was not explained by motion-insensitive orientation adaptation or attention. Taken together, the results suggest a trajectory-specific adaptable process that depends on higher-order representations after the integration of position and motion signals and after interhemispheric integration.

## Results

### Experiment 1: Trajectory aftereffect and interhemifield transfer

The effect of adaptation to motion trajectories with either a subjective or physical adapt-tilt was examined within either the ipsilateral or contralateral test hemifield relative to the adapted hemifield (the left or right hemifield was counterbalanced across participants). The adapting stimulus consisted of eight Gabor patches traveling up and down in parallel but in asynchronous oscillation phases. Their trajectories had either a subjective or physical tilt in either a clockwise (CW) or counterclockwise (CCW) tilt angle with respect to the vertical axis. In the subjective adapt-tilt condition, as shown in Figure 1, the subjective tilt was produced by the double-drift illusion in vertically oscillating patches. In the physical adapt-tilt condition (not shown in Figure 1), the physical tilt was produced by inclining the trajectories by the angle perceptually matched to the subjective tilt each participant would see in the double-drift illusion (see STAR Methods). The test stimulus was a luminance blob traveling downward along a trajectory with a variable tilt. The participants were asked to indicate whether the trajectory of the blob appeared as tilting CW or CCW. To quantify the effect, the point of subjective equality to the vertical axis was determined by fitting a logistic curve to the CW response proportions against the tilt of the test stimulus’s trajectory.

**Figure 2:**
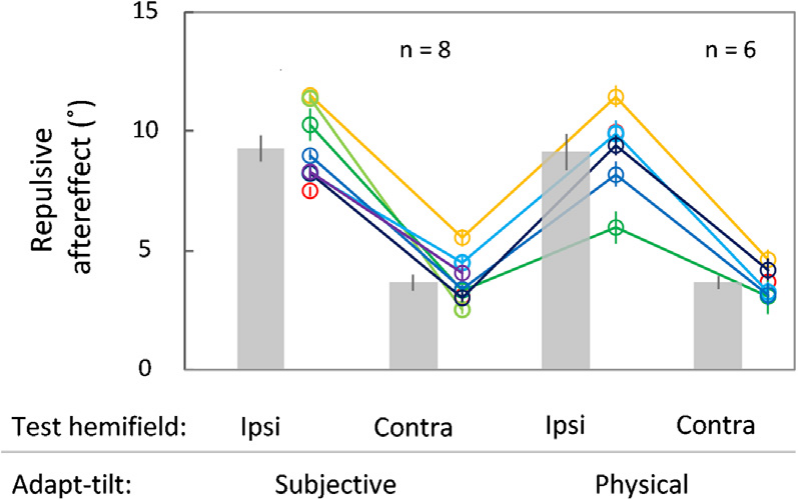
Results of the trajectory aftereffect in the subjective and physical adapt-tilt conditions, combined with the ipsilateral and contralateral test hemifields relative to the adapted hemifield (Experiment 1) The ordinate indicates the repulsive aftereffect size, defined as half the difference in subjective equality between the CW and CCW adapt-tilt conditions. The adapt-tilt was either subjectively available as the double-drift illusion or physically rendered to match the percept of the double-drift illusion. Individual and mean results are shown by circles of different colors and by bars, respectively. Error bars represent SE; for individual data, the SE was determined with 10,000 bootstrap iterations.

For all conditions, the point of subjective equality indicated that the perceived trajectory in the test stimulus traveling vertically was inclined repulsively from the vertical axis against the adapt-tilt angle (Figure 2; *M* = 9.30°, *SE* = 0.55°, one-sample t test, *t*_(7)_ = 17.01, *p* < 0.0001, *d* = 6.02, Bayesian one-sample t test, *BF*_10_ > 10,000 for subjective adapt-tilt and ipsilateral test; *M* = 3.67°, *SE* = 0.34°, *t*_(7)_ = 10.84, *p* < 0.0001, *d* = 3.83, *BF*_10_ = 1556.22 for subjective adapt-tilt and contralateral test; *M* = 9.15°, *SE* = 0.77°, *t*_(5)_ = 11.89, *p* < 0.0001, *d* = 4.86, *BF*_10_ = 304.73 for physical adapt-tilt and ipsilateral test; *M* = 3.66°, *SE* = 0.26°, *t*_(5)_ = 14.35, *p* < 0.0001, *d* = 5.86, *BF*_10_ = 626.63 for physical adapt-tilt and contralateral test; *n* = 8). This repulsive trajectory aftereffect was larger for the ipsilateral than for the contralateral test hemifield (main effect in a two-way repeated-measures analysis of variance [ANOVA], *F*_(1, 5)_ = 666.08, *p* < 0.0001, η_p_^2^ = 0.99, Bayesian repeated-measures ANOVA, *BF*_10_ > 10000; *n* = 6), and the aftereffects after adaptation to a subjective and physical tilt were not significantly different (main effect in the ANOVA, *F*_(1, 5)_ = 0.01, *p* = 0.93, η_p_^2^ = 0.002, *BF*_10_ = 0.37; interaction between adapt-tilt and test hemifield, *F*_(1, 5)_ = 0.03, *p* = 0.88, η_p_^2^ = 0.005, *BF*_10_ = 0.46; *n* = 6) after a listwise exclusion of the missing data from the two participants who did not take part in the physical adapt-tilt condition. To take account of the missing data, all the statistical results in the ANOVA were further corroborated by a generalized linear mixed model, with an identity link function using JASP software,^22^ where the response variable (aftereffect size) follows a Gaussian probability distribution. The adapt-tilt, test hemifield, and their interaction were used as fixed-effects factors, and participant was used as a random-effects factor (χ^2^_(1)_ = 0.00057, *p* = 0.98 for adapt-tilt; χ^2^_(1)_ = 25.37, *p* < 0.0001 for test hemifield; χ^2^_(1)_ = 0.02, *p* = 0.90 for interaction).

The aftereffect averaged >9° in the ipsilateral and >3.5° in the contralateral test hemifield. These two sizes of the aftereffect are comparable to a retinotopically confined static tilt aftereffect and a non-retinotopic static tilt aftereffect observed for complex patterns, respectively.^23^ The aftereffect we found cannot be explained by local motion adaptation to the drifting carrier per se, as its motion direction was periodically reversed, and any local motion adaptation was balanced between the symmetric directions. In addition, the test stimulus did not spatially overlap with the Gabor patches comprising the adapting stimulus in the contralateral condition. Therefore, our results suggest that, unlike motion aftereffects, the adaptation depended entirely on higher-order representations after the integration of position and motion signals, as the size of the subsequent aftereffect did not significantly differ, irrespective of whether the adapt-tilt was physically present or only subjectively available as the double-drift illusion. Also, the aftereffect would partially depend on an integrative process with little hemifield specificity, as almost half the original aftereffect size transferred between the left and right hemifields.

### Experiment 2: Perceived rather than physical trajectories determine the aftereffect

**Figure 3:**
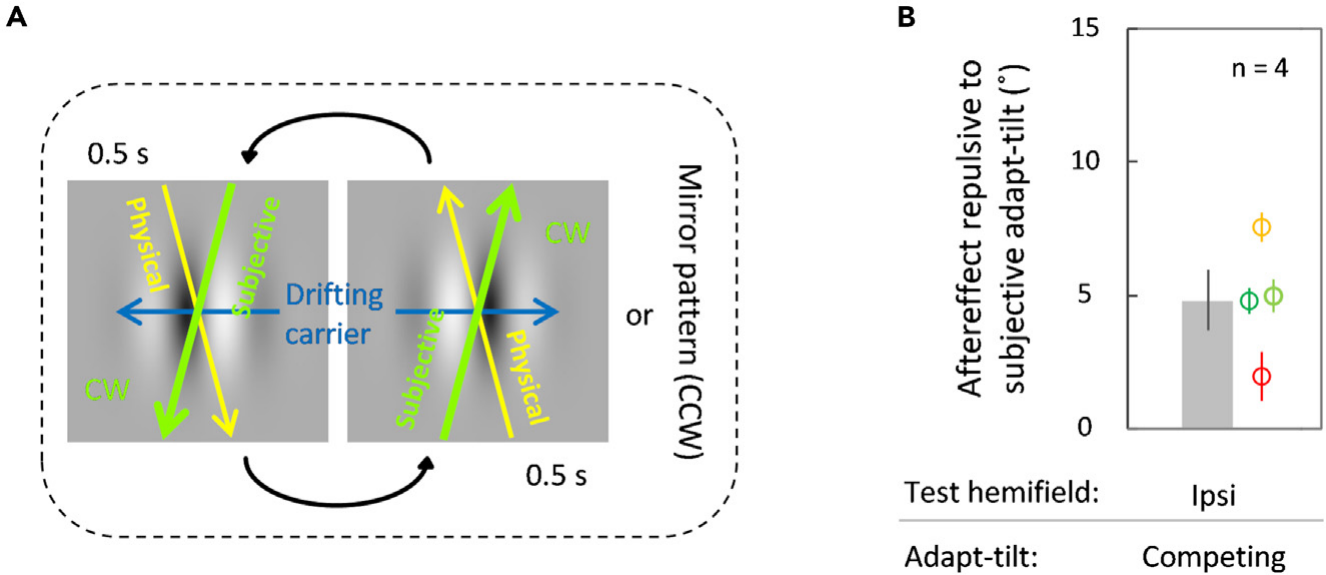
Stimulus configuration and results in the competing adapt-tilt condition (Experiment 2) (A) With respect to the vertical axis, the trajectory had a physical tilt on the opposite side to the subjective tilt seen in the double-drift illusion. The physical tilt from the vertical axis was half the double-drift illusion size for each participant, so that the subjective and physical tilts were balanced about the vertical axis. (B) The ordinate indicates the aftereffect, with a positive value indicating the angle from the vertical orientation against the subjective adapt-tilt. Other conventions are the same as those in Figure 2.

In Experiment 1, we found no differences in the aftereffect sizes in the subjective and physical adapt-tilts, suggesting that perceived trajectories explain the observed effect. To directly compare the contributions of the subjective and physical adapt-tilts, we used an adapting stimulus in which the physical tilt rendered on the screen and the subjective tilt seen in the double-drift illusion were “competing” with each other (Figure 3A). The physical tilt angle for each participant was calibrated prior to the aftereffect measurements, so that the physical tilt was mirror symmetrical with the subjective tilt with respect to the vertical axis. The methods were otherwise identical to Experiment 1, except that only the ipsilateral hemifield was tested.

The aftereffect was repulsive against the subjective adapt-tilt (Figure 3B; *M* = 4.82°, *SE* = 1.14°, one-sample t test, *t*_(3)_ = 4.21, *p* = 0.02, *d* = 2.11, Bayesian one-sample t test, *BF*_10_ = 3.86; *n* = 4). The results support the idea that the trajectory aftereffect is determined by subjective rather than physical tilt. As the size of the aftereffect was modest compared to that found in the ipsilateral conditions in Experiment 1, the potential contribution of physical tilt should not be ignored. However, this modest aftereffect might have occurred because the subjective adapt-tilt angle had to be halved to implement this special configuration (see STAR Methods) or because participants were different.

### Experiment 3: No evidence for the contribution of a motion-insensitive orientation process

It has been proposed that orientation information available as visible persistence can be used to determine the perceived object trajectory.^12^^,^^13^ It is technically possible for the perceived trajectory to have an influence in the opposite way, with the representation of an object’s trajectory tilt altering the computation of orientation-specific mechanisms that are insensitive to motion per se.^24^ To assess the contribution of such a motion-insensitive orientation process, which can be a high-level one in the processing hierarchy,^25^ the aftereffect was measured using a flashed stationary bar with a variable tilt, instead of a traveling luminance blob, as the test stimulus.

**Figure 4:**
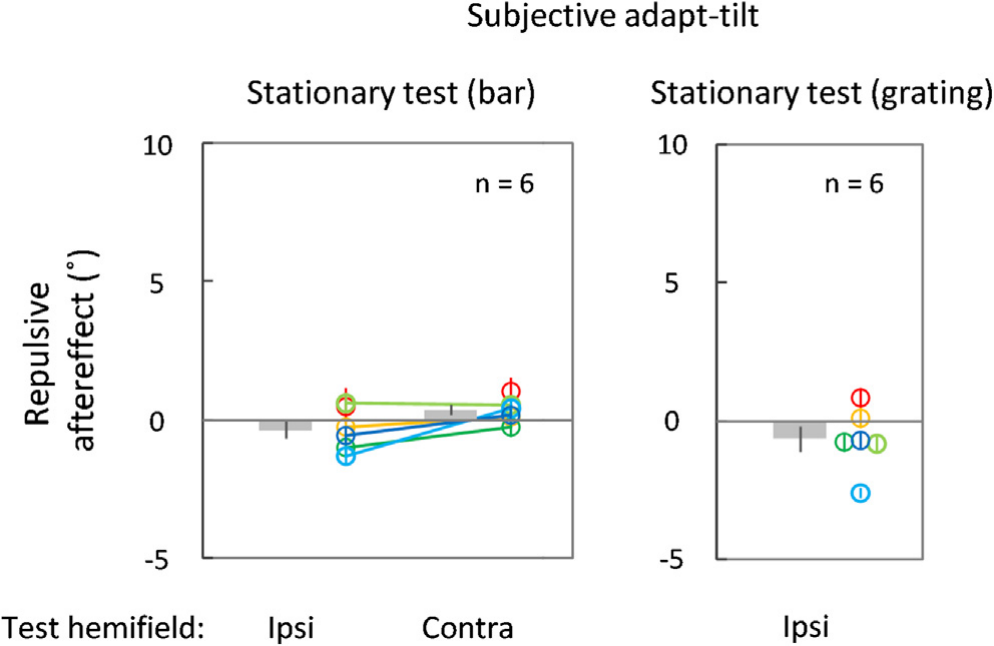
Results obtained under the stationary test conditions, combined with the ipsilateral and contralateral test hemifields The adapt-tilt was only subjectively available as the double-drift illusion. The test stimulus was a stationary bar (Experiment 3A) or a stationary sinusoidal grating (Experiment 3B) presented for 50 ms; the tilt of the test stimulus was manipulated in the same way as the tilt of the test trajectory in Experiments 1 and 2. Other conventions are the same as those in Figure 2.

The aftereffect was around zero and statistically insignificant for the ipsilateral (left panel of Figure 4; *M* = −0.36°, *SE* = 0.32°, one-sample t test, *t*_(5)_ = −1.13, *p* = 0.31, *d* = −0.46, Bayesian one-sample t test, *BF*_10_ = 0.69; *n* = 6) and for the contralateral test hemifield conditions (left panel of Figure 4; *M* = 0.33°, *SE* = 0.18°, *t*_(5)_ = 1.82, *p* = 0.13, *d* = 0.74, *BF*_10_ = 1.07; *n* = 6). To examine the potential role of stimulus differences from Experiments 1 and 2 as a confounding factor, we measured the aftereffect using a flashed stationary grating as the test stimulus.^24^ However, again, no aftereffect was statistically supported (right panel of Figure 4; *M* = −0.66°, *SE* = 0.47°, *t*_(5)_ = −1.39, *p* = 0.22, *d* = 0.44, *BF*_10_ = 0.74; *n* = 6). Interestingly, some of the participants exhibited an aftereffect on the attractive side (i.e., on the same side as the adapt-tilt), which might have been an illusion-dependent case of serial dependence.^26^ Although our adapting stimulus might have induced some adaptation of orientation-specific mechanisms, given the small aftereffect sizes and inconsistencies across participants, these data on the attractive side provide no evidence for the adaptation of a motion-insensitive orientation process. Instead, these findings suggest that a trajectory-specific process plays a major role in the trajectory aftereffect.

### Experiment 4: No evidence for the contribution of an attention process

Attentional tracking supports the localization of traveling objects^27^ and can influence motion perception,^28^^,^^29^ the process of which is subject to adaptation.^30^ As attentional tracking has limited capacity and hemifield-specific resources,^31^ the trajectory aftereffect and the interhemifield transfer of this aftereffect are not consistent with the characteristics of attentional tracking. Nonetheless, to assess the potential role of the attention process in the experimental paradigm in the present study, we measured the trajectory aftereffect with and without an additional attentional task during adaptation. In one of two conditions (hereafter “additional task-absent” condition), the participants were asked only to perform the same tilt-judgment task as in Experiment 1. In the other condition (“additional task-present” condition), the color of the fixation point changed rapidly during the adaptation period, and the participants were asked to count the times a target color (blue) appeared throughout the adaptation periods in each session^32^^,^^33^ and to perform the same aforementioned tilt-judgment task.

**Figure 5:**
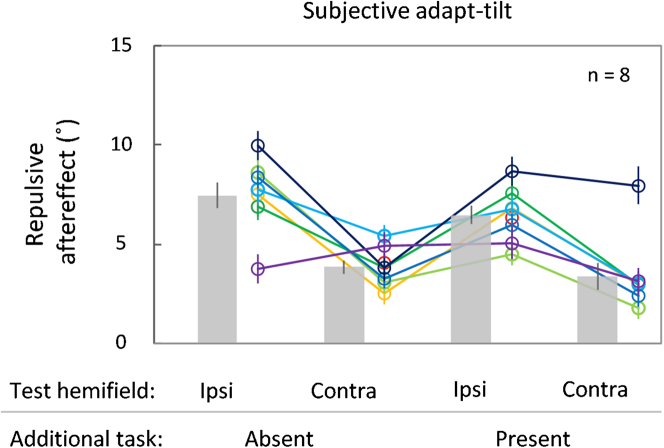
Results of the trajectory aftereffect in the additional task-absent and task-present conditions, combined with the ipsilateral and contralateral test hemifields (Experiment 4) The additional task required participants to count the number of times the blue target color appeared during adaptation. The adapt-tilt was only subjectively available as the double-drift illusion. Other conventions are the same as those in Figure 2.

The counting error was low (*M* = −1.51, *SD* = 5.93, total of 102.21 target presentations), suggesting that the additional task imposed some level of attentional and memory load and partly distracted the participants from attentional tracking. The repulsive aftereffect was significant for all conditions (Figure 5; *M* = 7.46°, *SE* = 0.64°, one-sample t test, *t*_(7)_ = 11.70, *p* < 0.0001, *d* = 4.14, Bayesian one-sample *t*-test, *BF*_10_ = 2387.28 for the additional task-absent and ipsilateral test; *M* = 3.86°, *SE* = 0.34°, *t*_(7)_ = 11.36, *p* < 0.0001, *d* = 4.02, *BF*_10_ = 2022.62 for the additional task-absent and contralateral test; *M* = 6.47°, *SE* = 0.47°, *t*_(7)_ = 13.67, *p* < 0.0001, *d* = 4.83, *BF*_10_ = 5785.19 for the additional task-present and ipsilateral test; *M* = 3.40°, *SE* = 0.67°, *t*_(7)_ = 5.09, *p* = 0.001, *d* = 1.80, *BF*_10_ = 30.97 for the additional task-present and contralateral test; *n* = 8). The repulsive aftereffect was larger for the ipsilateral than for the contralateral test hemifield (main effect in a two-way repeated-measures ANOVA, *F*_(1, 7)_ = 89.03, *p* < 0.0001, η_p_^2^ = 0.88, Bayesian repeated-measures ANOVA, *BF*_10_ > 10000; *n* = 8), and this difference occurred regardless of the absence or presence of the additional task during adaptation (main effect in the ANOVA, *F*_(1, 7)_ = 2.63, *p* = 0.15, η_p_^2^ = 0.27, *BF*_10_ = 0.52; interaction between the additional task and test hemifield, *F*_(1, 7)_ = 0.30, *p* = 0.60, η_p_^2^ = 0.04, *BF*_10_ = 0.45; *n* = 8). The results favor the null hypothesis, providing no evidence for the contribution of attention.

## Discussion

Together, our psychophysical experiments on the trajectory aftereffect suggest that there is an adaptable process specific to object trajectory. The findings that the aftereffect was equivalent for the subjective and physical adapt-tilts and occurred even when the subjective tilt was competing with the physical tilt suggest that adaptation involves higher-order position representations. This aftereffect is qualitatively different from the adaptation based on integrative motion signals, such as stereoscopic motion,^34^ non-retinotopic motion,^35^ and global plaid motion;^36^ these previous studies have focused on the motion/direction aftereffect that is perceived as repulsive against the direction of the adapting stimulus, whereas our aftereffect manifests itself as a perceptually altered trajectory orientation after adaptation to bidirectional diagonal motions. Furthermore, the transfer of the trajectory aftereffect between hemifields differs from the location specificity of classic motion aftereffects. In previous studies, the motion aftereffect partially transferred to retinally unadapted neighboring locations when moving patterns occupying large fields were used for adapting and test stimuli (a remote or phantom motion aftereffect^6^), and the motion aftereffect partially transferred even between the left and right hemifields when an expansion pattern was used.^5^ However, interhemifield transfer did not occur when a translation pattern was used.^5^^,^^37^ As spatial transfer is thought to reflect the receptive field sizes of neurons in the processing pathway, which progressively become larger at higher stages in the neural hierarchy, the trajectory aftereffect is likely to involve higher-order processes than those responsible for motion aftereffects.

We did not find any attentional modulation of the trajectory aftereffect. Recent psychophysical studies suggest that the double-drift illusion is constructed before attentional selection or tracking can operate.^38^^,^^39^ Adaptation could have the same bottleneck (i.e., dependence on perceived rather than physical trajectory) as attention does, so that attention processes and working memory can only access the perceived trajectories and positions.^38^^,^^40^^,^^41^ The present study suggests that hard-wired and hence adaptable units tuned to trajectory orientation are activated in an integrative processing stage prior to attention processes. Gain controls of these units through adaptation may shift the population tuning and repulsively alter the appearance of trajectory orientation, essentially in a manner similar to that proposed for motion and tilt aftereffects.^42^ Functionally, the alteration may be a consequence of efficient coding of the most dominant tilt angle for which the maximum discrimination sensitivity is achieved by shifting the population tuning of the activated units.

The involvement of the neural representation of the double-drift illusion and the partial interhemifield transfer discussed previously suggest that possible neural loci responsible for the trajectory aftereffect are located at integrative processing stages. Neurophysiologically, the dorsal medial region of the medial superior temporal area (MSTd) processes complex motion patterns, such as expansion and rotation over a wide field,^43^ with large neuronal receptive fields up to approximately 50 degrees of visual angle (hereafter “deg”).^44^ In contrast, the lateral ventral region of the medial superior temporal area (MSTl) processes small objects traveling over a wide field.^45^ A recent study reported that the neuronal activity in the ventral intraparietal area (VIP), rather than the MSTl, represents the trajectory of traveling objects in a more flexible manner with respect to the (head- and world-centered) reference frames.^46^ Furthermore, a functional magnetic resonance imaging study localized the neural correlates of the double-drift illusion in anterior cortical areas, including the prefrontal cortex (PFC).^47^ Given the large receptive fields, the sensitivity to object motion, and the relevance to the double-drift illusion, object trajectory may be adaptively processed in high-level regions, such as the MSTl and VIP. In these regions, feedback signals from even higher-level regions, such as the PFC, could be involved, without the involvement of hemifield maps.

There are only a few studies on the role of adaptation in object trajectory perception.^13^^,^^14^ The lack of studies might be due to object trajectory perception being considered a special case of motion perception of patterns consisting of a few moving elements (as in our adapting stimulus that consisted of multiple patches). However, the adaptation paradigm used in the present study reveals a distinct computational process for trajectory perception. As trajectory processing has potential benefits for the biological visual system, its properties and neural mechanisms should be the subject of future investigations.

### Limitations of the study

The present study has two methodological limitations. First, the potential involvement of static tilt aftereffects cannot be fully refuted based on the absence of an effect of trajectory adaptation on the stationary test stimuli. In the stationary test conditions, the shape modifications in the test stimulus from a blob to a bar and grating are potential confounders. This limitation should be addressed by systematically varying the spatiotemporal properties of test stimuli in future research. Second, the task difficulty may have affected the findings relating to attentional modulation. In the additional task-present condition, the manipulation of attentional and memory loads might not have been sufficient to influence the trajectory aftereffect. Although the same additional task has been used in previous studies,^32^^,^^33^ this limitation should be addressed in future research by using a more difficult additional task before drawing firm conclusions about the independence of the trajectory aftereffect from attention.

## STAR★Methods

### Key resources table

**Table:** 

REAGENT or RESOURCE	SOURCE	IDENTIFIER
**Deposited data**		

Data and original code	This paper	Figshare (https://doi.org/10.6084/m9.figshare.24265876.v2)

**Software and algorithms**		

MATLAB	Mathworks Inc.	https://www.mathworks.com/products/matlab.html
Psychophysics Toolbox	Brainard (1997)^48^; Pelli (1997)^49^	http://psychtoolbox.org/
Vision Toolbox	Nakayama & Motoyoshi (2018)^50^	https://park.itc.u-tokyo.ac.jp/motoyoshilab/VTB.html
Palamedes Toolbox	Prins & Kingdom (2018)^51^	https://www.palamedestoolbox.org/
JASP	JASP Team (2019)^22^	https://jasp-stats.org/

### Resource availability

#### Lead contact

Further information and requests for resources should be directed to and will be fulfilled by the lead contact, Ryohei Nakayama (ryohei.nakayama.ac@gmail.com).

#### Materials availability

This study did not generate new unique reagents.

#### Data and code availability

• Data have been deposited at the Figshare and are publicly available as of the date of publication. Accession number is listed in the key resources table.
• All original code has been deposited at the Figshare and is publicly available as of the date of publication. Accession number is listed in the key resources table (same as the accession number of the data).
• Any additional information required to reanalyze the data reported in this paper is available from the lead contact upon request.

### Experimental model and study participant details

Eight human observers (four females) participated in the subjective adapt-tilt condition, six of whom (three females) also participated in the physical adapt-tilt condition (Experiment 1). Another four observers (two females) participated in the competing adapt-tilt condition (Experiment 2). Another six observers (one female) participated in the stationary test condition with the bar stimulus (Experiment 3A). Another six observers (two females) participated in the stationary test condition with the grating stimulus (Experiment 3B). Another eight observers (six females) participated in the additional task-present and task-absent conditions (Experiment 4). All observers were Japanese and between the ages of 18 and 22. The samples were of convenience, and the sample sizes were determined before the data collection. Based on preliminary observations, we expected a large effect in the order of approximately 0.5 with low variance, indicating that four or five participants should be sufficient to achieve high statistical power.

All participants had normal or corrected-to-normal vision and provided written informed consent. All experiments were conducted in accordance with the Declaration of Helsinki (2003) and approved by the ethics committee of the Graduate School of Humanities and Sociology at the University of Tokyo.

### Method details

#### Apparatus

In all experiments, images were displayed on a gamma-corrected 22-inch cathode ray tube (CRT) screen (800 × 600 pixels) with a frame rate of 75 Hz (Iiyama A201H initially and after sudden failure, Iiyama HM204DA for Experiments 1 and 4; Mitsubishi RDF223H for Experiments 2 and 3). The images were generated on a computer using MATLAB (Mathworks Inc.), Psychophysics Toolbox,^48^^,^^49^ and Vision Toolbox.^50^ The CRT resolution was 4.6 min/pixel at a viewing distance of 37 cm constrained by a chin rest for binocular viewing.

#### Stimuli

A black fixation dot (0.4 deg in diameter) was presented at the center of the screen on a uniform gray background (59.2 deg wide and 45.4 deg high; 42.4 cd/m^2^ or 40.0 cd/m^2^ for Experiments 1 and 4; 50.1 cd/m^2^ for Experiments 2 and 3). The adapting stimulus consisted of eight Gabor patches, four above and four below the horizontal meridian (Figure 1). Each Gabor patch was a vertical sinusoidal grating (spatial frequency: 1.5 cycles/deg; peak contrast: 0.99), contrast-modulated according to a Gaussian function (SD: 0.8 deg). The center of the presentation area for the array of the Gabor patches was randomized across trials between 14.9 deg and 17.2 deg horizontally from the fixation point. The adapting stimulus was presented in the left hemifield for half the participants and in the right hemifield for the other half.

The vertical (or diagonal in the physical adapt-tilt condition in Experiment 1 and Experiment 2) back-and-forth movements of the Gabor patches were mutually asynchronous, with constraints being that the phase difference between each adjacent pair of patches should be greater than 45° and that they should always be 6.0 deg horizontally and >3.0 deg vertically apart from each other. The positional oscillation was 1 cycle/s with a traveling speed of 16.5 deg/s. In the experiments in which the double-drift illusion was induced, the vertical carrier of each Gabor patch drifted horizontally at 5.5 deg/s either to the left or to the right, reversing the direction synchronously with the back-and-forth movements of the patch, whereas in the physical adapt-tilt condition in Experiment 1, the vertical carrier did not move horizontally.

In Experiments 1, 2, and 4, the test stimulus was a blob with an isotropic Gaussian luminance profile (SD: 1.6 deg; peak contrast: 0.5) traveling at 11 deg/s for 0.5 s. In Experiment 3A, the test stimulus was a stationary bar (4.7 deg wide and 19.0 deg high with each edge blurred by a 2.4-deg-wide raised cosine function; contrast: 0.5). In Experiments 3B, the test stimulus was a stationary sinusoidal grating (spatial frequency: 2.7 cycles/deg; contrast: 0.2; SD of an isotropic Gaussian envelope: 4.7 deg). The physical tilt of the test stimulus was manipulated according to the method of constant stimuli per adapt-tilt angle. There were six tilt levels with 30 trials per level for each condition of Experiment 1, and seven levels with 24 trials per level for each condition of the other experiments. In addition, other tilt levels were introduced in the subjective adapt-tilt condition in Experiment 1 for three participants who showed large biases in subjective equality. The center of the test stimulus was 16.1 deg horizontally, either to the left or to the right of the fixation point. The ipsilateral and contralateral hemifields were tested for the same number of trials in random order within a session in Experiments 1, 3A, and 4.

#### Procedure

The experiments were conducted in a dark room. The CW and CCW adapt-tilts were presented with an 8-min inter-session interval or on separate days. The order of the adapt-tilt angles (combined with the additional task-present and task-absent conditions in Experiment 4) was counterbalanced across participants. On each day, the experiment was performed in six sessions after practice, in addition to the prior calibration if applicable (the physical adapt-tilt condition in Experiment 1 and Experiment 2). Each session consisted of 56–60 trials, with a 2-min break at the end of each session.

Throughout each session, the participants were asked to maintain their gaze on the fixation point at the center of the screen. In each trial, the adapting stimulus was presented for 5 s. In the first trial of each session, the adapting stimulus was presented for 30 s, followed by a 50 ms pause, and then it was presented again for 5 s. After an interstimulus interval of 50 ms, the test stimulus was presented for 0.5 s (50 ms in Experiments 3A and 3B). After the test stimulus had disappeared, the participants indicated whether the perceived tilt of the test stimulus was CW or CCW by pressing a button. There was no feedback as to whether the response was correct or incorrect. If they did not respond within 2 s, a beep sound informed them of trial abortion, and the next trial was started.

For calibration prior to the physical adapt-tilt condition in Experiment 1, the amount of physical tilt was perceptually matched to the illusory tilt for each participant using a two-interval forced choice procedure. First, it was introspectively reported that a Gabor patch traveling along a trajectory with a physical tilt indeed appeared to move along this physical tilt when its carrier was stationary with respect to the envelope. Second, an array of these control Gabor patches and the stimulus used for the subjective adapt-tilt condition in Experiment 1, which traveled vertically but induced an illusory tilt, were presented one by one for 1 s each in a random order, with an interstimulus interval of 0.5 s. The participants judged in which interval the trajectories had a more CW tilt. The physical tilt of the control Gabor patches was varied over 60 trials by a staircase with a step size of 4° and a one-up, one-down rule to home in on a 50% proportion of “interval with the physical-tilt trajectory” responses. The point of subjective equality to the vertical axis was determined as the tilt corresponding to the 50% proportion by fitting a logistic curve with the maximum likelihood method.

For calibration prior to Experiment 2, the amount of physical tilt was perceptually matched to the illusory tilt for each participant in the same way as the prior calibration in the physical adapt-tilt condition in Experiment 1. In the main sessions of Experiment 2, the adapting stimulus was the same as that used in the subjective adapt-tilt condition in Experiment 1, except that the physical tilt was added in the opposite way to the subjective tilt by half the double-drift illusion size quantified as the perceptually matched tilt angle in the prior calibration, so that the subjective and physical tilts were balanced about the vertical axis.

In the additional task-present condition in Experiment 4, the added task demanded attention and working memory. Thus, the participants were asked to focus on rapid color changes of the fixation point. During the adaptation period, the color changes between red, green, blue, yellow, purple, and cyan occurred randomly at a rate of 2 colors/s. The fixation point remained black during the other periods including the test period. The participants were instructed to count the number of times the blue color appeared throughout the successive adaptation periods within each session.

### Quantification and statistical analysis

For each condition, we estimated the point of subjective equality corresponding to a 50% proportion of CW responses by fitting a logistic curve with the maximum likelihood method, using the Palamedes Toolbox.^51^ The repulsive aftereffect size was defined as half the difference in the point of subjective equality between the CW and CCW adapt-tilt angles. The aftereffect sizes were then compared against zero by one-sample *t*-tests and across conditions by an ANOVA. Statistical analyses were performed using JASP statistical software.^22^ The statistical details of experiments can be found in the Results section. No methods were used to determine whether the data met assumptions of the statistical approach.
